# Historical Aspects of Endemic Trachoma in Peru: 1895–2000

**DOI:** 10.1371/journal.pntd.0004116

**Published:** 2016-01-14

**Authors:** Vicente Maco, Mayling Encalada, Carlos Wong, Luis A. Marcos

**Affiliations:** 1 Institute of Tropical Medicine Alexander von Humboldt, Universidad Peruana Cayetano Heredia, Lima, Peru; 2 Ministry of Public Health, Quito, Ecuador; 3 Institute of Ophthalmology Wong, San Isidro, Lima, Peru; 4 Departments of Medicine, Microbiology and Molecular Genetics, Stony Brook University, Stony Brook, New York, United States of America; Alfaisal University, SAUDI ARABIA

## Introduction

Trachoma, a chronic keratoconjunctivitis caused by the intracellular bacterium *Chlamydia trachomatis*, is the leading infectious cause of blindness and affects the most underprivileged populations worldwide. An estimated 1.3 million people have been blinded, and there are more than 50 trachoma-endemic countries, mainly in Africa, the Middle East, and Asia [1,2].

In Latin America, countries such as Brazil, Guatemala, and Mexico have targeted the areas where trachoma has been identified, according to the Report on the Sixteenth Meeting of World Health Organization (WHO) Alliance for the Global Elimination of Trachoma by 2020 (GET 2020) [3]. Recently, Colombia has reported pockets of trachoma among indigenous populations in Vaupés, [4] San Joaquín, and Santa Catalina, and it is identifying additional areas for completeness of the data [5]. Other countries, such as Peru, Bolivia, and Venezuela, which are likely to be endemic, according to these sources, have not reported population-based estimates. This lack of trachoma assessment at the population level was noted and put on the agenda of the WHO GET 2020, a target that could not be reached without recent and well-designed studies in suspected countries.

Regarding Peru, the current status of trachoma is unknown. A search of the two main e-libraries, the Medical Literature Analysis and Retrieval System Online (MEDLINE) and the Scientific Electronic Library Online (ScIELO), using keywords such as trachoma, trichiasis, and Peru, produced no articles. Only one database considered as gray literature, the Latin American Literature in the Health Sciences (Literatura Latinoamericana en Ciencias de la Salud, or LILACS), produced seven articles, all in Spanish and not referenced in any large recent reviews on blindness, trachoma, or other neglected tropical diseases in Latin America and the Caribbean. Moreover, the full versions of these articles were not available, even after institutional request.

To fill this gap, we have conducted an extensive and critical historical review of the local literature and have searched all available sources of early written documentation and pictorial evidence of trachoma in Peru.

## Historical Review of the Local Literature

### Search strategies

As a first search strategy, we screened the main electronic medical databases, which included MEDLINE, ScIELO, Embase, Web of Science, and Scopus, along with gray-literature resources such as LILACS, Google Scholar, Google Books, and OpenGrey. We used the following search terms and phrases in combination and in any order: “trachoma,” “trichiasis,” “follicular conjunctivitis,” “entropion,” “blindness,” “*Chlamydia trachomatis*,” “chlamydozoos,” “inclusion bodies,” “corpuscular inclusions,” “intracellular inclusions,” “Halberstaedter-Prowazek bodies,” “Latin America,” “South America,” and “Peru.” We searched for sources in English, Spanish, or French, without restriction of publication date. For all sources retrieved, we hand-searched all references cited at the end of the publication that were relevant to the main topic. We excluded sources whose data were incomplete (lacking the place of origin of the study or including unsupported statements or erroneous citations), minimal, duplicated from previous studies by the same working group, null on trachoma epidemiology in Peru, or from a different country as a result of a mismatch. Publications by authors who worked on trachoma and lived in Peru but did not generate any data on trachoma during their stay in the country were excluded as well [6,7]. We also reviewed the personal collections of ophthalmology experts in the field to cover journals, local meetings, and any other personal reports.

Sources that were found during this first approach and that were not available online were recovered directly from one of the authors or coauthors or from institutional medical libraries in two countries: the National Institute of Ophthalmology and the Museum for the Health Sciences, Major National University of San Marcos (Museo de Ciencias de la Salud de la Universidad Nacional Mayor de San Marcos—UNMSM), both located in Lima, Peru, and the National Institute for Public Health Research (Instituto Nacional de Investigacion en Salud Publica—INSPI), located in Guayaquil, Ecuador. The types of historical sources were exhaustive and included non-indexed, non-peer-reviewed, ephemeral, and local journals, doctoral theses, books, dissertations, meetings, conference reports, bulletins, pamphlets, and conference proceedings. An ephemeral journal was defined as any scientific journal published at any time during a certain period, usually short (less than 50 years), but whose publication was discontinued or inactive by the time we started our search (2013). Local doctoral theses were screened using the following database repositories: the Peruvian Network of Digital Thesis (www.rptd.edu.pe) and two others from the major universities in Lima: Cayetano Heredia University (www.upch.edu.pe/vrinve/publicaciones/) and the National University of San Marcos (UNMSM) (http://cybertesis.unmsm.edu.pe/). Given that parts of certain data reports were published twice in the same or in different journals, we thoroughly scrutinized all tables and graphics and recalculated prevalence percentages whenever possible to avoid repetition of data.

Given the limited data, our second search strategy was the inclusion of the Rare Book and Manuscript Collection of the Center for the History of Medicine and Public Health, the historical medical library of the New York Academy of Medicine, located in New York, New York, United States (http://nyamcenterforhistory.org), and founded in 1847. We included this library because it is considered to be one of the largest archives of medical collections in the US, including original Peruvian journals and other documents from the early part of the century that are not available elsewhere. Since the most recent reports from WHO (2012) [3] and the Pan American Health Organization (PAHO; 2011) [5] in the Americas were available by the time we began our search, the neglected infectious diseases department of PAHO was not contacted.

### Classification of historical sources

The historical sources were classified as (i) direct evidence (any case series, case reports, or population surveys on trachoma performed by the main author(s) listed in the source), (ii) indirect evidence (any secondhand reference to case series, case reports, or populations surveys on trachoma by an author not listed in the original source and different from the main author or coauthors), or (iii) pictorial evidence (any drawing, photograph, or other type of graphic reproduction of any stage of trachoma, such as follicular trachoma, intense trachoma, scarring, trichiasis, or corneal opacity, published in the sources or shared by local authors contacted in Peru).

All historical sources were tabulated according to their references, year of publication, authors, source, study setting (hospital, outpatient clinic, or population survey), region (community or district), survey methodology (retrospective, case series, or cross-sectional), diagnostic method (clinical, cytology, or cell culture), total population, number and percentage of definitive cases, trachoma signs, and period of study (year[s] of duration of the study). We applied the 1987 WHO simplified grading system for the assessment of trachoma [8] based on the presence of follicles (TF: trachomatous inflammation—follicular), pronounced inflammatory thickening (TI: trachomatous inflammation—intense), or scarring (TS: trachomatous scarring) in the upper tarsal conjunctiva; presence of one eyelash rubbing the eyeball (TT: trachomatous trichiasis); or visible corneal opacities over the pupil (CO: corneal opacity) wherever possible. When none of these trachoma signs could be found (only four sources [9–12] were published after the WHO grading system was developed in 1987), we categorized them as unspecified signs of trachoma (U).

Individuals’ origin, age, and sex were recorded when available. The setting of each document was categorized as follows: hospital (reports from hospitalized individuals explicitly mentioned by the author), population survey (reports from “eye campaigns,” defined as free-of-charge screening surveys led by ophthalmologists that included evaluation of visual acuity, fundoscopy, external eye examination, eyelid eversion, and, in some cases, Gram stain and cytology of the tarsal conjunctiva and/or surgical interventions, over a non-calculated number of individuals usually evaluated on a first-come, first-served basis in a determined period of time [usually less than 30 days] and place), and outpatient clinic (reports from individuals seen by an author in his or her private practice and/or office). Although the number of individuals was not calculated before the beginning of most of the studies, except in one [12], it usually comprised around 80% of the population living in these small communities (<1,000 individuals), according to the authors. When the setting of the document (individuals’ origin) was not specified anywhere in the main text, we categorized it as non-defined (ND). According to the Peruvian National Institute of Statistics and Informatics (Instituto Nacional de Estadística e Informática—INEI), a community is a group of people composed of families that inhabit and control a specific territory and that are linked by social, cultural, and economic interests. A district is defined by the INEI as a minor geopolitical division in a country; it is divided into urban and rural areas. A timeline of all of the sources describing cases of trachoma was generated using the visual processor SmartDraw version 2012 (San Diego, California, US). Each source was tabulated according to the date of publication (month/day/year) in a yearly timeline view. A map showing the geographical locations (by department and district) of the most recent studies (those performed after 1980) was generated with the same visual processor and using geographic coordinates when available at the INEI. For simplification, only those studies that reported more than ten individuals with any sign of trachoma were included in the map.

Finally, due to the limited number of sources that were recovered during our search, all of the materials, including non-indexed articles, doctoral theses, conference proceedings, book chapters, dissertations, and photographs of evidence of trachoma by local investigators, were manually scanned and converted into a portable document file (PDF) for future access (see supplemental files). All selected material appropriate to the topic was included with the permission of the author(s) or copyright holder(s).

## Ethical Considerations

The study was approved by the local Ethics Committee of the National Institute of Ophthalmology (NIO) in Lima, Peru.

## Results of the Historical Review

**Fig 1:**
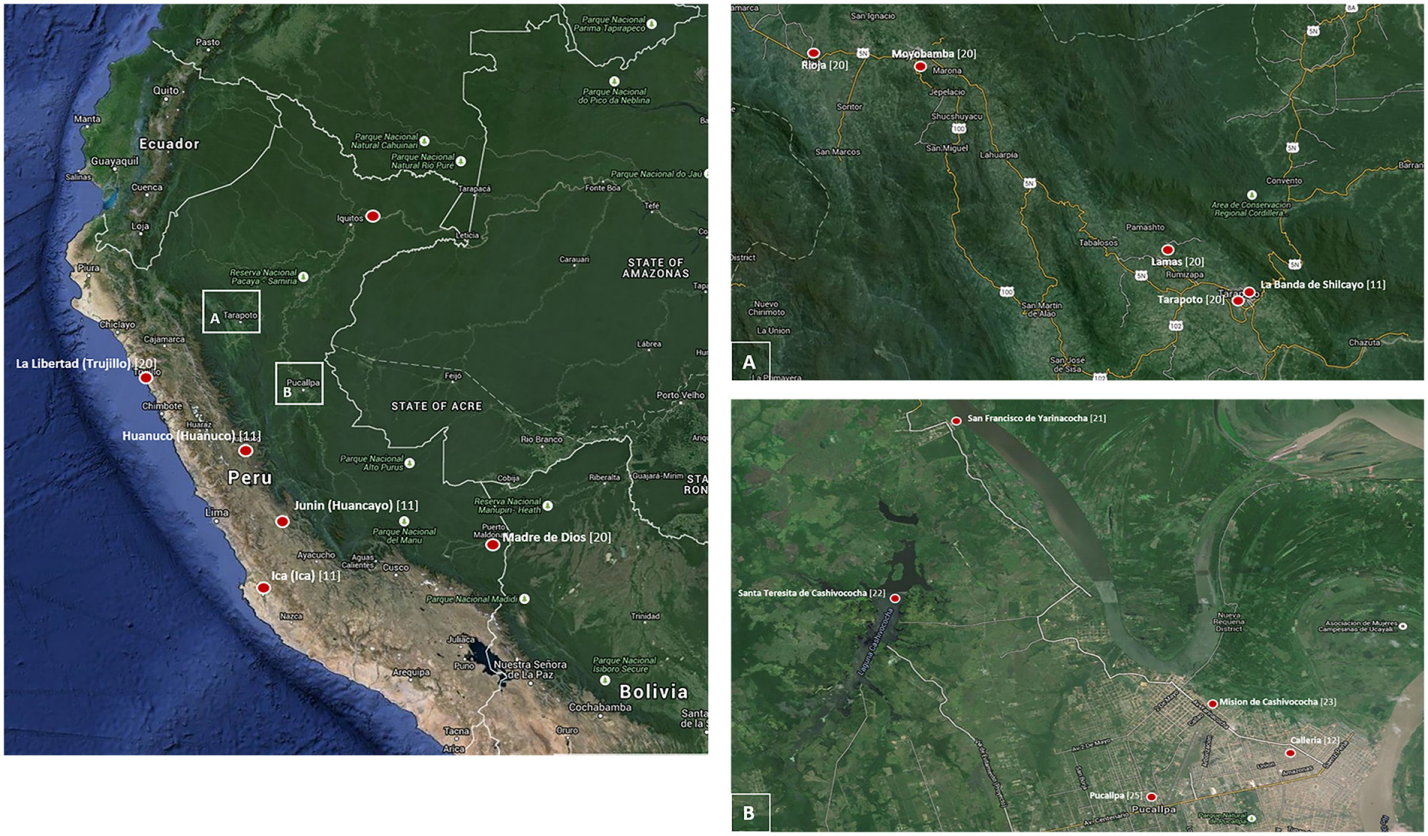
Map of Peru with geographical areas where trachoma was reported after the 1980s. Only included are the studies that reported more than ten individuals with any sign of trachoma. Areas (o) in the main map denote the department of Peru, followed by the district or community in parentheses and its reference in brackets. (A) Magnification of the department of San Martin showing five districts or communities and references in brackets. Surveys performed between 1983 and 2000. (B) Magnification of the department of Ucayali showing five districts or communities and references in brackets. Surveys performed between 1985 and 2001.

A total of 66 historical sources (36 journal articles, 18 photographs, three meeting reports, two book chapters, two theses, one bulletin, one study report, one conference proceeding, one Peruvian Ministry of Health technical report, and one dissertation) matched our first strategy criteria. Of these, we excluded 50 sources (38 written sources and 12 photographs) that did not meet our inclusion criteria. Thus, a total of 16 documents (12 journal articles, two doctoral theses, one dissertation, and one book chapter) that were published over a period of 105 years, specifically from 1895 to 2000, registered the presence of local and imported trachoma in Peru (Table 1). Among these sources, only one is indexed and available in a major electronic medical library [13]. All of these sources were written in Spanish and published in local journals by Peruvian editors. Of all photographs provided by one coauthor (Carlos Wong [CW]), six were selected as pictorial evidence. A map of Peru showing the communities in which trachoma has been reported after the 1980s is shown in Fig 1. This map only includes the studies that reported more than ten individuals with any sign of trachoma. Areas in the main map denote the department of Peru, followed by the district or community in parentheses and its reference in brackets.

**Table 1:** Year of publication, author, source, setting, region survey methodology, diagnostic method, total population, percentage of cases, and period of study of sources of early documentation of endemic and non-endemic trachoma in Peru: 1895–2000.

Ref.	Year of publication^§^	Author	Source	Setting^¶^	Region^∑^ (community or district)	Survey methodology	Diagnostic method ^∫^	Total population^£^	Cases (%)	Trachoma signs^∞^					Period of study
										TF/TI	TS	TT	OC	U	
[14]	1895	Gaffron, E.	Journal	ND	Lima	Retrospective	C	3,247	23 (0.7)	14	-	9	-	-	1893–1895
[15]	1918	Burga, B	Journal	H^Ω^	Lima	Retrospective	C	NA	67 (NA)	-	-	-	-	67	1910–1918
[16]∂	1935	Campodónico, E.	Dissertation	C	Lima	Retrospective	C	42,949	179 (0.4)	-	-	-	-	179	1911–1934
[17]	1942	Navarro, L. F.	Thesis	H^Ω^	Lima	Retrospective	C	424	14 (3.3)*	8	8	6	-	-	1939–1942
[18]	1954	Galvez, J. (I)	Thesis	ND	ND	Case series	C	ND	61 (NA)	-	-	-	-	61	ND
[19]	1960	González del Río, A.	Book	C	Madre de Dios	Retrospective	C	ND	162 (NA)	-	-	-	-	162	1955–1959
[20]	1983	Tenorio, A. et al (I)	Journal	P	Madre de Dios (Maldonado, La Cachuela, La Pastura, Tres Islas, El Pilar, La Joya, Lago Valencia, Puerto Prado, El Castañal)	Retrospective	C	1,005	452 (44.9)	-	-	-	-	452	1955–1956
				H^Ω^	La Libertad	Case series	C	ND	12 (NA)	-	-	-	-	12	1976
				P	San Martin (Tarapoto, Moyobamba, Lamas, Rioja, Escuela)	Cross-sectional	C	555	125 (22.5)	125	-	-	-	0	1976
[21]	1985	Tenorio, A. et al	Journal	P	Ucayali (San Francisco de Yarinacocha)	Cross-sectional	C, Cy	102	88 (86.2)	-	-	1	-	87	1983
[22]	1986	Wong, C et al	Journal	P	Ucayali (Santa Teresita)	Cross-sectional	C, Cy	408	302 (74.0)	302	-	-	-	-	1985
[23]	1986	Tenorio, A. et al	Journal	P	Ucayali (Misión de Cashivococha)	Case series	C, Cy	NA	7 (NA)	6	1	-	-	-	1985
[24]	1986	Tenorio, A. et al	Journal	P	Loreto (Manatí II)	Case series	C, Cy	142	54 (38.0)	45	-	-	-	9	1985
[25]	1987	Tenorio, A. et al	Journal	P, C, H^Ω^	Lima (Comas, Canto Grande, Independencia, San Martin de Porras, San Luis, Pueblo Libre, La Molina)	Case series	C, Cy	ND	85	-	-	-	-	85	ca. 1985–1986
					La Libertad (San Pedro de Lloc)										
					Loreto (Iquitos)										
					Junín (La Merced, San Ramón)										
					Ucayali (Pucallpa)										
					Ayacucho (Mala)										
[9]	1995	Wong, C	Journal	P, C	Lima	Retrospective	C	15,479	238 (1.5)	-	-	-	-	238	1989–1993
[10]	1996	OPELUCE^ψ^	Journal	C	Ancash (Huaraz)	Retrospective	C	323	1 (0.3)	-	-	1	-	-	1995
[11]	2000	Arévalo, L. F.	Journal	P	Lima, Ica, Junín, Huánuco, San Martin	Retrospective	ND	23,057	20 (0.1)	-	-	-	-	20	1989–2000
[12]	2001	Wong, C. et al	Journal	P	Ucayali (Calleria)	Cross-sectional	C	924	172 (18.6)	-	165	7	-	-	2000

^§^ Year of documentation;

^¶^ H: hospital, P: population survey, C: outpatient clinic, ND: non-documented.

^∫^ C: clinical, Cy: cytology.

^£^ NA: non-applicable.

^∞^According to the WHO Grading System of Trachoma: trachomatous inflammation—follicular (TF), trachomatous inflammation—intense (TI), trachomatous scarring (TS), trachomatous trichiasis (TT), and corneal opacity (CO). U: unspecified signs of trachoma in the original text;

^ψ^ Organización Peruana de Lucha contra la Ceguera (Peruvian Organization Against Blindness)

^∑^ Community and district definitions according to the INEI.

^Ω^ No reason for the individuals’ hospitalization was specified in any of the reports.

^∂^ In his dissertation, Campodónico presents cases of trachoma seen in the outpatient clinic at the Italian Hospital of Lima in the period 1911–1934. Notice that Burga’s series (1910–1918) from the same hospital included eight years (1911–1918) that may have posteriorly been added in Campodónico’s publication. Three years of this Burga-Campodónico overlap (1913, 1915, and 1917) report the same number of trachomatous patients. See text for further explanation.

* Navarro screened 424 clinical records, 14 of which were from trachomatous patients. Eight additional cases of trachoma were included from other sources, finally reporting 22 cases. See text for further explanation.

### Direct and indirect evidence

**Fig 2:**
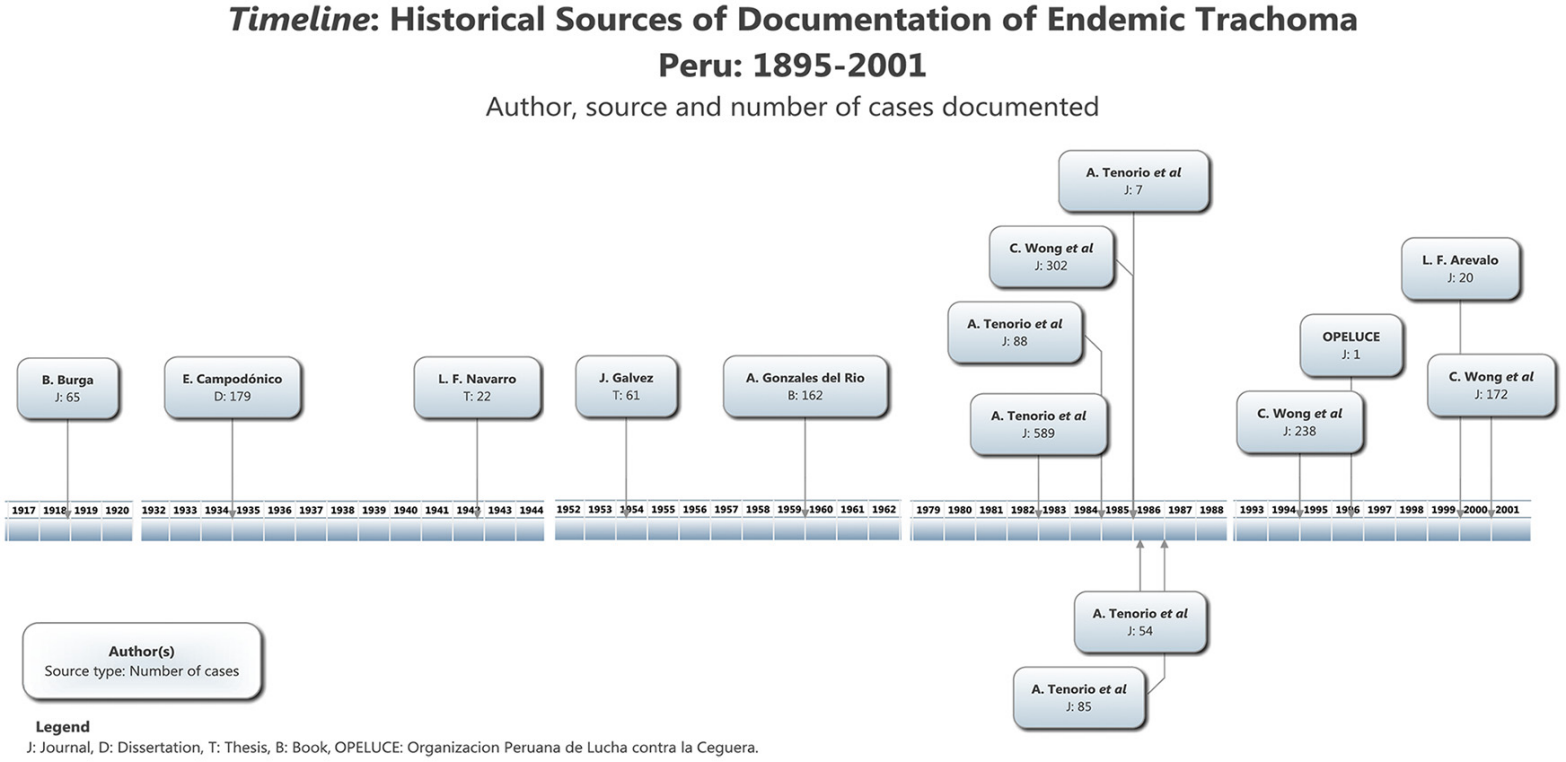
Timeline. Dates of publication of different sources of trachoma in Peru.

The publications (*n* = 16) were deliberately divided into two periods (Fig 2).

(1) Period I, from 1895 to 1960 (6/16): Most of the sources (4/6) from this period represent retrospective studies or case series performed in outpatient clinics and hospitals located in Lima (Ophthalmological Clinic of the Italian Hospital Vittorio Emanuel II, Archbishop Loayza National Hospital, and 2 de Mayo Hospital) [14–17]. The exceptions (2/6) are the doctoral thesis *The Socio-Medical Problem of Blindness in Our Environment* (El Problema Medico-Social de la Ceguera en Nuestro Medio), by Jose Galvez [18], in which no setting or region was documented, and the book *Five years as a physician in Madre de Dios* (Cinco años de médico en el Madre de Dios), by Arturo Gonzales del Rio, in which he narrated his years of experience working in the southern rainforest of Madre de Dios [19].

During this period, the total number of individuals screened was 46,620, of whom 506 had any form of trachoma. None of these sources is currently indexed. Two (references [14] and [19]) were only accessible by appointment at the Center for the History of Medicine and Public Health, New York Academy of Medicine, New York, US. Five sources were considered as direct historical evidence according to our classification criteria.

(2) Period II, from 1983 to 2000 (10/16): Most of these sources (9/10) represent population surveys performed in the indigenous and non-indigenous populations of Ucayali (5) [12,21–25], Lima (3) [9,11,25], Loreto (2) [24,25], La Libertad (2) [20,25], Madre de Dios (1) [20], San Martin (2) [11,20], Junín (1) [25], Ancash (1) [10], Huánuco (1) [11], Ayacucho (1) [25], and Ica (1) [11]. These field studies, with the exception of the one headed by L. F. Arévalo (1/17) in 2000, were conducted by founding members of the nongovernmental organization (NGO) the Peruvian Organization Against Blindness (Organizacion Peruana de Lucha contra la Ceguera—OPELUCE).

During this period, the total number of individuals screened was 41,995, of whom 1,556 had any form of trachoma. Only four sources were indexed in LILACS. Most of them (6/10) were retrievable from the same author (CW) and were not available elsewhere.

### Results by period

Period I was predominantly characterized by case series and doctoral theses of cases that presented in the capital Lima, with some of the individuals from other parts of the country; whereas period II was distinguished by the “eye campaigns” performed in aboriginal communities in the rainforest and occasional reports of trichiasis in the highlands.

#### Period I

The presence of endemic trachoma in Peru was documented by local ophthalmologists as early as 1893 [14]. The earliest record of trachoma appeared in the original article “Analytic statistics based on 3247 ophthalmopathies observed in Lima” (Estadistica analitica basada en 3247 oftalmopatias observadas en Lima), written by Eduardo Gaffron in 1895 [14]. This article was published in *La Crónica Médica*, a biweekly journal of medicine, surgery, and pharmacy. These patients were observed by the author between 1893 and 1895. He evaluated the eyelids of 348 patients (11.72%) and found nine cases of trichiasis and 14 of ophthalmia granulosa (what he later calls trachoma). Although the term “trachoma” was not listed in his statistics, he mentioned these 14 cases of trachoma (0.43%) later, in the section “Ophthalmia Granulosus” (Oftalmia granulosa) of his manuscript, reporting a total of 23 cases of trachoma (TT + TF). Age ranges of individuals included were not available.

In 1918, Buenaventura Burga presented a total of 67 cases between 1910 and 1918 from the Italian Hospital Vittorio Emanuel II [15], a hospital founded by the Beneficiary Italian Society of Lima in 1881. Burga’s original article, also published in *La Crónica Médica*, could not be found, so critical information from this report was indirectly collected from *Bibliographic Summaries* (Extractos Bibliograficos) [26], appearing in the Cuban Journal of Ophthalmology, Volume I, page 367, in 1919, and from the *Dictionary of Peruvian Medicine* (Diccionario de Medicina Peruana) [27], by H. Valdizan, partially published in the Annals of the School of Medicine, Volume 43, Number 4, page 77, in 1960. Unfortunately, age range of these 67 cases was not specified in either publication.

In 1935, in his dissertation *Trachoma in Peru* (El tracoma en el Perú), Esteban Campodónico reported 179 (0.41%) cases of trachoma from a total of 42,949 patients evaluated between 1911 and 1934 in the Ophthalmological Clinic of the Italian Hospital in Lima [16]. In contrast to Burga, who reported cases from the same institution from 1910 to 1918 [15,26,27], Campodónico included 16 additional years, from 1919 to 1934. Regarding the overlapping years of study of the two authors, only 1913, 1915, and 1917 had similar numbers of trachomatous patients: six, nine, and eight, respectively. Additionally, in the section of his dissertation entitled “Topography of trachoma in Peru” (Topografia del trachoma en el Peru), he mentioned that trachoma predominated in Ica and that he had observed cases from Loreto (rainforest), but he did not provide concise data on epidemiology. Age ranges of individuals included were not provided by the author.

In 1942, Luis F. Navarro presented his medical doctoral thesis, *Monograph of trachoma*, *as a contribution to its study in Peru* (Monografia del tracoma, como contribucion a su estudio en el Perú), and described 22 cases of trachoma [17]. He retrospectively reviewed 424 medical charts from two hospitals in Lima, Archbishop Loayza National Hospital and 2 de Mayo Hospital, finding 14 cases of trachoma (seven from each hospital) from 1939 to 1942. He added eight cases referred by other physicians to his series. From this total of 22, 12 were considered as cases of local trachoma, and ten were considered as imported (nine Asians and one Italian who arrived in Peru with trachoma). The age range of this series was 13–65 (12 males, ten females). Similar to Campodónico [16], in Navarro’s thesis’ section “History and geographical distribution” (Historia y distribucion geografica), he stated that ophthalmologists frequently reported cases of trachoma from Loreto, without other supporting epidemiological evidence.

In 1954, Jose Galvez presented his medical doctoral thesis, *The Socio-Medical Problem of Blindness in Our Environment* (El Problema Medico-Social de la Ceguera en Nuestro Medio), in which he cited the lecture *Trachoma in Peru* (El trachoma en el Peru), by Jorge Valdeavellano [18]. This lecture was presented at the II Pan American Meeting of Ophthalmology in Montevideo, Uruguay, in 1945. In the thesis section called “General Opinion about Trachoma in Peru” (Opinion general sobre el trachoma en el Peru), Galvez refers to Valdeavellano’s lecture, adding 61 cases of trachoma, 34 of which were in natives of Peru. The period of the study, age ranges of the individuals, and the setting were not documented.

In 1960, Arturo Gonzales del Rio, a Spanish physician who arrived in Peru in 1951, published the book *Five years as a physician in Madre de Dios* (Cinco años de médico en el Madre de Dios), reporting a total of 162 cases of trachoma in the area [19]. The cases were from outpatient clinic records and were retrospectively collected from 1955 to 1959. No data on the age range or sex of individuals were provided. In his chapter “Pathology of the Area” (Patologia de la zona), trachoma represented the fourth most common diagnosis (*n* = 162), only after intestinal parasitosis (*n* = 422), bronchitis (*n* = 228), and malaria (*n* = 191).

#### Period II

No other source of documentation could be located until 1983, when Arturo Tenorio and colleagues reviewed the national literature from 1955 to 1983, reporting (a) one retrospective study by Trujillo and colleagues (1955–1956) that described 452 trachoma cases from Madre de Dios, from a total of 1,005 school-aged children whose age range was not specified or stratified; (b) one cases series by Sanchez de Caceda and colleagues (1976) that reported a total of 12 cases of trachoma from La Libertad (age range not specified); and (c) one cross-sectional study by Silverio and colleagues (1976) that reported a total of 125 cases of follicular conjunctivitis compatible with trachoma from a total of 555 individuals examined from San Martin (age range not specified) [20].

**Fig 3:**
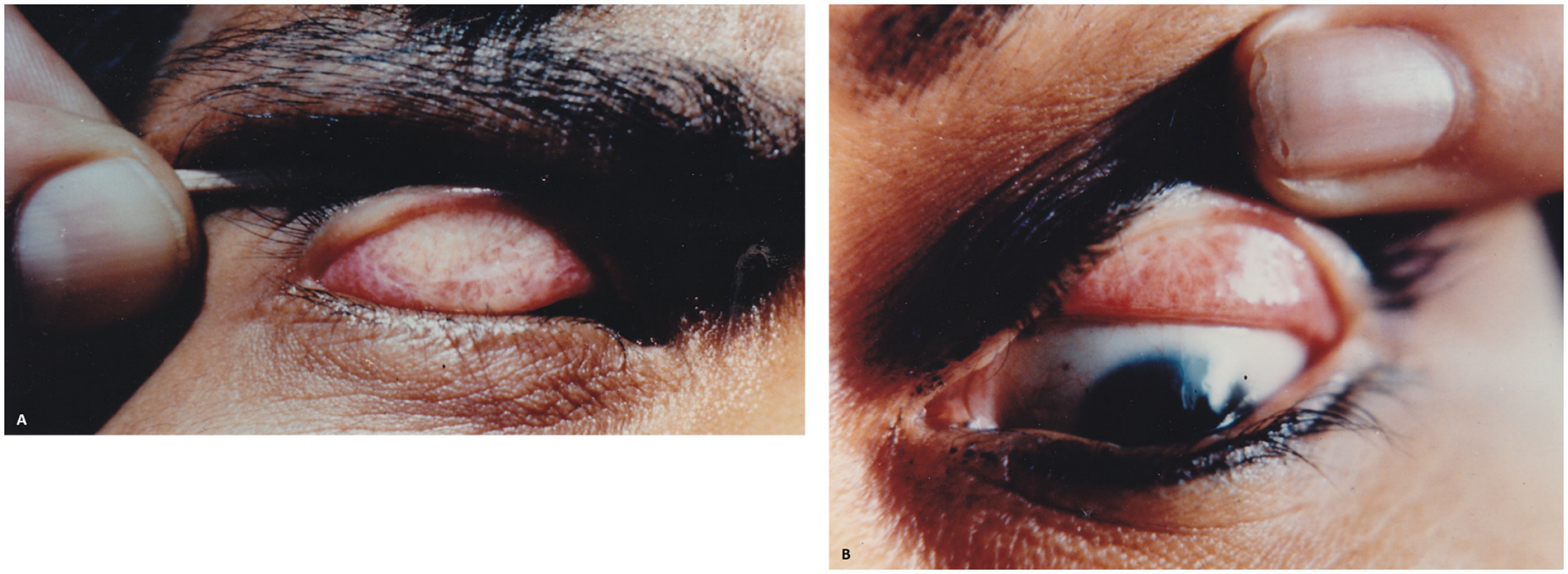
Pictorial evidence of endemic trachoma in Peru. (A) Everted upper eyelid showing scarring trachoma (TS) in an individual from San Francisco de Yarinacocha in 1985 [21] and (B) in another individual from Santa Teresita in 1986 [22], two indigenous Shipibo-Conibo communities settled along the banks of the Ucayali river, Pucallpa, department of Ucayali. The field studies were performed from 1983 to 2001 and were led by one of the authors (CW).

In September of 1983, Arturo Tenorio, leading a group of Peruvian ophthalmologists gathered by one of the authors (CW) in 1979, founded OPELUCE [28–30], an NGO that performed the first field study in an Amazonian aboriginal community in Peru [21]. Their results were published two years later, in 1985, in the *Peruvian Journal of Ophthalmology* [21]. This community comprised approximately 500 Shipibo-Conibo individuals from San Francisco de Yarinacocha, Ucayali (Fig 3), 102 of whom were evaluated for any ocular diseases (age range of the studied population: three to 15 [*n* = 11], 16 to 30 [*n* = 77], and greater than 30 [14]), with 88 cases of follicular conjunctivitis suggestive of trachoma being found (age range of the trachoma cases was not provided). Of note, they also performed Giemsa stain-based cytology of the upper conjunctival tarsus on ten slides, all of which had inclusion bodies.

In 1985, one of the authors (CW) and colleagues, as part of a series of subsequent health campaigns, performed another population survey in the Shipibo-Conibo community of Santa Teresita, Ucayali (Fig 3) [22], which was published in 1986. Of the 408 individuals examined, the researchers found 302 cases of follicular conjunctivitis and 19 cases of tarsal scarring. Even though the authors did not provide age ranges, the majority of the population was between ten and 30 years old and was predominantly female, according to their report. In addition, they performed cytological studies on 38 slides and found that 94.73% had inclusion bodies.

Between 1985 and 1986, Arturo Tenorio and colleagues reported a case series of seven Swiss missionaries (aged 10–46; six males, one female) living among indigenous people in Mision de Cashivococha, Ucayali (1985) [23]; an outbreak of follicular conjunctivitis that affected 54 school-aged children (142 total children examined, aged 6–11) living in the locality of Manatí, situated along the banks of the Amazon river, in Loreto (1985) [24]; and finally, a case series of 85 patients with trachoma (age range and sex not provided) from urban and marginal urban areas of Lima, La Libertad, Loreto, Junín, Ucayali, and Ayacucho (1986) [25]. Giemsa stain-based cytology was performed in all of these studies.

In 1995, one of the authors (CW) published a retrospective study in which he described the Integral Model of Care for Ocular Health (Programa de Atencion Integral de Salud Ocular—PAISO): primary ocular health care, community sanitary education, and clinical and surgical specialized eye care [9]. This report included 15,479 patients from marginal urban areas of Lima (1989–1993) and 57,212 patients from rural areas of the rest of the country (1983–1993). In the marginal urban areas, 238 patients with trachoma (among other ocular pathologies) were recorded. The individuals’ origin was grossly recorded as one of the three main geographical regions of Peru: coast, highlands, or rainforest. No age range, sex, or a more specific origin (district, community, province, or department) was provided in the original tables or text.

OPELUCE presented the results of their PAISO activities (clinical evaluation of eye pathologies, cataract surgeries, visual acuity, and intra- and post-surgical complications) from 1994 to 1995 in Chiclayo, Huaraz, Huancayo, Pucallpa, and Lima (Villa El Salvador) in a publication in 1996 [10]. Of 323 individuals from their eye campaigns, only one case of trichiasis, from Huaraz, department of Ancash, was registered. No age range or sex was provided.

In 2000, Luis F. Arévalo, director of the Ophthalmic Service, a private group of ophthalmologists who had worked toward the local prevention of blindness since 1989, presented his group’s experience divided into two stages [11]: one that comprised the period of 1989–1997 and was performed in urban and marginal urban areas of Lima, Huancayo, Ica, Huánuco, Tarapoto, and Chancay; and a second one that comprised the period of 1998–2000 and was conducted in Lima, Callao, and Huancayo. The number of individuals assessed between 1989 and 2000 was 20,573; among them, a total of 20 trachoma cases in urban Lima (one of 10,251), marginal urban Lima (two of 6,406), the highlands (eight of 2,027), and the forest (nine of 894) were found. No age, sex stratification, or specific origin (except from geographical region as described above) was provided or tabulated.

Lastly, in 2000, one of the authors (CW) and colleagues performed a descriptive, cross-sectional epidemiological study in a marginal urban area of Coronel Portillo Province (district of Calleria) in Ucayali [12]. The sample (*n* = 1,114) was calculated on the estimates of blindness in the population (~0.7%). They evaluated 924 individuals (age ranges 0–14: 225; 15–44: 399; 45–59: 186; and greater than 60: 114), most of them mestizos, 165 of whom had scarring trachoma and seven of whom had trichiasis. The age and sex of these individuals with scarring trachoma and trichiasis were not documented in their report. Among other ocular pathologies, scarring trachoma and trichiasis represented the fourth (17.9%) and the tenth (0.8%) most common diseases in this population. This study represented the last epidemiological survey referring to trachoma performed in Peru, and there have not been any other estimates since then.

### Pictorial evidence

Regarding the pictorial evidence, 18 photographs were collected from one of the authors (CW), six of which depicted different clinical signs of trachoma and laboratory evidence of the presence of *C*. *trachomatis*:

1. One cytology photomicrograph (1) that showed inclusion bodies, and one cell culture (1) positive for *C*. *trachomatis* infection (*n* = 2).
2. Three photographs that showed scarring trachoma (one from the community of San Francisco de Yarinacocha and one from Santa Teresita) and one additional picture that revealed trichiasis on an elderly female from a Campa-Ashaninka community in La Merced, Junín) (*n* = 4).

## Discussion

In contrast to recent reviews on blindness and trachoma in Latin America, in which epidemiological data on Peru are scanty, limited, or absent [31–37], we have found a notable number of sources (*n* = 16) that report the presence of any stage of trachoma in up to 2,062 patients evaluated by different ophthalmologists over more than a century (107 years), with the earliest report made by E. Gaffron in 1895 [14]. The disparity in the numbers of sources found in this study and in other reviews is likely due to the fact that the historical documents are not indexed in current libraries’ search engines and that all of these documents were published in Spanish in local specialized journals. In addition, we did not establish a time search criterion, and we included reports of trachoma in Peru at any time and in languages other than English. Interestingly, although several of these studies were performed in urban and marginal urban areas of Lima [9,11,25], most of them came from aboriginal communities located along the Amazon river [11,12,19–24] and reported a prevalence of any stage of trachoma as high as 94.9% [20]. Furthermore, contact with the ophthalmologists directly involved in these studies helped us to retrieve additional direct, indirect, and pictorial evidence of trachoma from these population studies. The additional use of three main libraries in different countries (Ecuador, Peru, and the US), including a historical medical repository, also contributed additional sources to our report.

During the first period (1895–1960), all early reports of trachoma in Peru were written by ophthalmologists settled in Lima, who communicated cases of trachomatous patients and considered the disease to be a rare occurrence in the country. E. Gaffron was the first author to present statistics on trachoma in Peru, in 1895. At the time, he referred to trachoma as “ophthalmia granulosa” and stated that “ophthalmia follicular” was not a sine qua non condition to develop the first form [14]. He also noted that the agent of gonococcal conjunctivitis was not the same as that of trachoma, as was believed by certain individuals at the time. As we will see, the ophthalmia granulosa of Gaffron was considered to be a singular disease by most of the authors in this first period [14–16,18].

In the following years (1910–1934), B. Burga and E. Campodónico described patients from the clinic in the Italian Hospital, and, despite the number of patients recorded from this one institution, the researchers regarded trachoma as an uncommon local disease, seldom observed in native Peruvians [15,16]. Interestingly, Hideyo Noguchi (1876–1928), a tropicalist who worked on *C*. *trachomatis* cultures in Ecuador at that time [7,38,39], visited E. Campodónico and had the opportunity to observe “classic chlamydozoos” (inclusion bodies) from patients in Lima in 1920 [16]. In the same period, L. F. Navarro was one of the first authors who, after an extensive description of the history and geographical distribution of trachoma, commented that the disease was being more commonly observed than before, even in certain areas such as the rainforest of Peru (Iquitos) [17]. Among the detailed descriptions of his 22 cases, there were seven cases of trichiasis, the late, blinding stage of trachoma, which suggests affected individuals have suffered repeated cycles of *C*. *trachomatis* infection over many years. Later, in 1954, J. Galvez considered acute neonatal conjunctivitis and trachoma to be the two most important transmissible ocular diseases in Peru and stressed the presence of endemic trachoma in Valdeavellano’s series. Furthermore, he described the importance of executing law 2348, dated September 19, 1924, which made the systematic notification of trachoma mandatory in Peru (to date, reporting of trachoma to the Ministry of Health is not regularly performed, despite the 1924 law) [18]. Six years later, A. Gonzales del Rio was the first physician who reported trachoma in the forest of Peru (Madre de Dios), ranking it as the fourth most common pathology observed during his years working as a physician in that area [19].

During the second period (1983–2001), the NGO OPELUCE, through volunteer health campaigns (“eye campaigns”), conducted most of the studies in the country and contributed to the documentation of trachoma and other eye pathologies in indigenous, forgotten populations remotely located in the Peruvian rainforest, reporting nearly 40,000 cases of trachoma [9,40–45]. This group of Peruvian ophthalmologists was founded in March, 1979 [28–30]; took care of 190 marginal urban populations, 70 of which were from the coast, highlands, and forest; examined 400,000 patients; and performed 14,000 eye surgeries [44,46,47]. In an attempt to generate a map of trachoma, A. Tenorio reviewed the local literature and reported the prevalence estimates of other authors (Trujillo and Lazarus) [20] who worked in Madre de Dios, with the highest percentage of infection in the locality of Lago Valencia (94.90%). He also reported the first population survey performed in aboriginal communities in the forest (the Shipibo-Conibo community of San Francisco de Yarinacocha, Ucayali) [21], the finding of trachoma among non-indigenous people living with natives (Swiss missionaries from Mision de Cashivococha, Ucayali) [23], an epidemic of follicular conjunctivitis among children (Manatí, Loreto) [24], and a summary of findings of trachoma in urban and marginal urban areas [25].

With a public health approach similar to what we now know as the SAFE strategy (surgery, antibiotics, facial cleanliness, and environmental improvement) [48,49], they applied their multidisciplinary PAISO model, which consisted of four aspects: primary clinical eye care, sanitary education for communities, and clinical and surgical specialized care [45,50]. The PAISO model was the result of the first campaigns for the prevention of blindness, which later oriented research programs that targeted the epidemiology of ocular diseases; operative aspects for the prevention of blindness, trachoma, xerophthalmia, and leprosy; and aboriginal communities in the rainforest [40]. From this experience, the group clearly recognized certain characteristics that appear to be constant regarding the epidemiology of trachoma in Peru: it is more prominent in the rainforest, it affects certain indigenous communities [41–43], and the predominant form is the non-blinding type [9,51], as it occurs in the Yanomami Indians living in the Brazilian Amazon [52]. Based on this data, which seems to be similar to other trachoma-endemic ethnic groups that lack primary health services and sanitation in the Americas (often settled in small, isolated groups, such as in Mexico [53,54], Guatemala [55], Colombia [4], and Brazil [32,52,56]), aboriginals may represent the population most vulnerable to endemic trachoma in Peru, and would represent a primary target to update the status of trachoma in the country.

Literature reviews about blindness, trachoma, and other neglected tropical diseases in Latin America have not included (or have partially included) Peru as a country with endemic trachoma [2,32–34,36,57]. Munoz and colleagues extensively reviewed the data on blindness and visual impairment in the Americas and the Caribbean [32]. Concerning the data from Peru, they included only three of the 16 sources found in our historical review. Although our study is a critical examination of any source of information on trachoma and, for that reason, did not apply any limitation to publication time or language, the data reported by these authors came from work performed relatively recently by OPELUCE in areas of high endemicity of trachoma in aboriginals (despite publication in non-peer-reviewed local journals). As we have previously mentioned, the data from these sources have to be critically and temporarily analyzed, given the limited methodology in reporting trachoma in these marginalized sectors of the population of Peru. Other literature reviews by Silva and colleagues [36] and Furtado and colleagues [57], who reported causes of blindness and other eye diseases and care in Latin America, succinctly described the problem of trachoma and suspected that the disease is also present in Bolivia and Peru. The dissimilarity of our findings, with more than 2,000 cases of trachoma in indigenous [12,19–22,24,41–43,51] and non-indigenous populations from different settings [14–18,23,25] dispersed over 33 urban, marginal urban, and remote communities from 12 of the 24 departments of Peru [9–11,25,40,44,45], is evident. These findings give us a better understanding of the history of trachoma in this country, placing the first cases of trachoma in or before 1893, which is much earlier than the beliefs of certain local authors, who dated the first cases in Peru to 1955 [9,20].

The data from the sources found during our search, although important pieces of information that had not been evaluated before and that were provided by experienced ophthalmologists, have certain limitations. Several of these data were not from population-based studies, but rather from hospitals, outpatient clinics, or case series, and these data may not reflect the real prevalence in those sectors of the country. From a public health perspective, standardized trachoma survey methods that have been developed, such as the Population-Based Prevalence Surveys (PBPS), Trachoma Rapid Assessment (TRA), and Acceptance Sampling Trachoma Rapid Assessment (ASTRA) [58–60], are methods of surveillance that have not been seriously applied to estimate the prevalence of trachoma in Peru. Of course, with inaccurate population-based data, the goal of mapping the global distribution of trachoma cannot be reached [61–63]. The development of this map, as showed by Polack and colleagues in 2005 [61], needs district-level estimates, which were not clearly defined in the Peruvian studies. However, the exclusion of this important information on Peru, most likely due to language barriers (all of the studies are in Spanish, and several are from sources such as Google Scholar and LILACS) or limited access to non-indexed local journals, is of concern for the global trachoma program, which we hope that this paper will help to begin to rectify. As we have observed during our extensive search, there have been repeated calls to provide information on the status of trachoma and trichiasis in certain Latin American countries [1,32,36,37,57,61–64], without further action from the health authorities and other organizations. Another characteristic of the sources is the lack of use of WHO’s simplified grading system in their assessment [8]. However, we believe that the assessment by ophthalmologists documented in the sources we summarize, though not prepared with reference to the WHO grading system, are sufficiently reliable to mandate contemporary evaluation of these endemic communities in the Amazon. Work to establish whether these populations remain trachoma-endemic is of critical importance to the current Global Trachoma Mapping Project and the program for the global elimination of trachoma as a public health problem, which it supports.

Finally, it is important to note that recent meetings about trachoma elimination in the Americas by WHO and its regional office, PAHO, have emphasized the need to provide updated information on trachoma in Peru and in other countries such as Bolivia and Venezuela. As we observed after this historical review, after the last population-based survey in 2001 [12], no more studies have been undertaken. Additionally, we have noticed certain misconceptions about *C*. *trachomatis* infection in Peruvian indexed journals, and that there are no recent local reports to the Ministry of Health, classifying trachoma as a neglected disease in this region. A standardized and well-conducted survey of trachoma among in-need and isolated populations is an important public health goal, given that there are groups of individuals living in remote areas where trachoma is highly prevalent, according to recent sources. Mapping the distribution of trachoma in Peru should be done soon for its public health importance. These studies are a priority for achieving the goal of recognizing forgotten pockets of diseases and represent our next step.

## Conclusions

Trachoma has existed in Peru, where this condition was recorded as early as 1893. The reports of trachoma extend over more than a century and include at least half of the regions of Peru. Our results help to expand our understanding of the history of trachoma in Peru. Prior to our literature review, the first cases of trachoma were believed to have been reported in 1955. Trachoma has affected aboriginal communities in the Peruvian rainforest, so population-based prevalence surveys in those communities with validated standardized methods are needed. The reports of this review were published more than ten years ago, and may not reflect the current situation. In Peru, trachoma is a forgotten disease and is not included in programs for blindness prevention. Although a law was created that mandates reporting trachoma, this is not implemented in practice. Important information regarding the status of trachoma in Peru has been excluded from previous extensive literature reviews in the Americas, in addition to a repeated call to update the data. Although the information collected from Peru is relevant, studies lack the rigorous methodology needed to report accurate estimates of the burden of the disease in this country.

## References

[1] Burton MJ, Mabey DC. The global burden of trachoma: a review. PLoS Negl Trop Dis. 2009;3(10):e460 10.1371/journal.pntd.0000460 19859534 PMC2761540

[2] Hotez PJ, Bottazzi ME, Franco-Paredes C, Ault SK, Periago MR. The neglected tropical diseases of Latin America and the Caribbean: a review of disease burden and distribution and a roadmap for control and elimination. PLoS Negl Trop Dis. 2008;2(9):e300 10.1371/journal.pntd.0000300 18820747 PMC2553488

[3] World Health Organization. Report of the Sixteenth Meeting of the WHO Alliance for the Elimination of blinding trachoma by 2020. Washington DC, US: 2012.

[4] Miller H, Gallego G, Rodriguez G. [Clinical evidence of trachoma in Colombian Amerindians of the Vaupes Province]. Biomedica: revista del Instituto Nacional de Salud. 2010;30(3):432–9. Epub 2011/06/30. . 21713345

[5] Organization PAH. Trachoma Elimination in the Americas—First Regional Meeting of Program Managers. Bogota, D.C.: PAHO, Regional Office of the WHO, 2011 5 23–25, 2011.

[6] Macchiavello A. El virus del tracoma y su cultivo en el saco vitelino del huevo de gallina. Revista Ecuatoriana de Higiene y Medicina Tropical. 1944;1:33.

[7] Cook G. Some less well-documented pioneers. Tropical Medicine: An Illustrated History of The Pioneers. Great Britain: Elsevier Science; 2007.

[8] Thylefors B, Dawson CR, Jones BR, West SK, Taylor HR. A simple system for the assessment of trachoma and its complications. Bulletin of the World Health Organization. 1987;65(4):477–83. 3500800 PMC2491032

[9] Wong C. Enfermedades Oculares y Ceguera en el Peru: Estudio epidemiologico. Arch peru oftalmol. 1995;7(1):10–44.

[10] Organizacion Peruana de Lucha contra la Ceguera (OPELUCE). Evaluacion de los Programas de Atencion Integral de Salud Ocular (PAISO) de OPELUCE. 1996 Contract No.: 1.

[11] Arévalo LF. Programas de Oftalmic Service y Experiencia en Namibia. Arch peru oftalmol. 2000;12:32–4.

[12] Wong C, Geronimo F, Morales D, Nava A, Bejarano L, Chin J, et al Prevalencia y causas de ceguera en la provincia de Coronel Portillo—Ucayali, Selva del Peru. Arch peru oftalmol. 2001;13:31–8.

[13] Muñoz M, Caballero P, Ayllón C, Medina S. Conjuntivitis folicular por Chlamydia trachomatis: Frecuencia y pruebas diagnósticas. Rev peru med exp salud publica. 2007;24(3):286–9.

[14] Gaffron E. Estadistica analitica basada en 3247 oftalmopatias observadas en Lima. La Crónica Médica. 1895;12(166):347–61.

[15] Burga B. [Trachoma in Peru]. Cronica Medica. 1918;35:360–6.

[16] Campodónico E. El tracoma en el Peru: Disertación presentada por el Dr. Esteban Campodónico al Congreso Medico Nacinal, convocado en homenaje a la celebración del IV centenario de la fundación de Lima. Lima, Peru: Tailleres Gráficos "Guia Lascano"; 1935.

[17] Navarro L. Monografía del tracoma como contribución a su estudio en el Perú. Lima: Universidad Nacional Mayor de San Marcos; 1942.

[18] Gálvez J. El problema médico-social de la ceguera en nuestro medio. Lima: Universidad Nacional Mayor de San Marcos; 1954.

[19] Gonzalez del Río A. Cinco años de medico en el Madre de Dios. Lima: Instituto de Estudios Tropicales Pío Aza—Litografia Universo; 1960 p. 143.

[20] Tenorio A, Siverio C, Contreras F. [Some aspects of trachoma clinic behavior in Peru]. Rev peru oftalmol. 1983;9(2):28–33.

[21] Tenorio A, Chávez F, Chea C, Guerra L, Tobaru L, Wong C, et al Tracoma en una comunidad Shipiba de la selva del Perú. Rev peru oftalmol. 1985;11(1):3–6.

[22] Wong C, Nava A, Calderón M, Guerra L, Chea C, Pongo L, et al Hallazgos oftalmológicos en la comunidad Shipiba de Santa Teresita: tracoma, demostración citológica. Rev peru oftalmol. 1986;12(1):14–6.

[23] Tenorio A, Wong C, Celiz E, Nava A. Sorprendente presencia de tracoma en misioneros suizos que laboran en la amazonia peruana. Rev per oftalmol. 1986;14(1):33–4.

[24] Tenorio A, Ardiles C, Calderon M, Chea C, Guerra L, Nava A, et al Epidemia de conjuntivitis folicular en la comunidad de Manati, en el rio Amazonas. Rev per oftalmol. 1986;12(2):23–6.

[25] Tenorio A, Wong C, Tobaru L, Orjeda O, Aguinaga O, Kuahara C, et al Tracoma en poblaciones urbano y urbano marginales del Perú. Arch peru oftalmol. 1987;2(1):26–7.

[26] Anonymus. Extraxtos bibliograficos. Revista Cubana de Oftalmología. 1919;I(1–2):367.

[27] Valdizan H. Diccionario de Medicina Peruana (T. VI, II parte). Anales de la Facultad de Medicina. 1960;XLIII(4):53–77.

[28] Montenegro E. OPELUCE: 20 Años Cuidando sus Ojos. Arch peru oftalmol. 1999;11(1):44.

[29] Wong C. La Historia. Arch peru oftalmol. 1999;11(1):45.

[30] Wong C. El diario intimo de la prevencion de la ceguera (Memorandum). Arch peru oftalmol. 1999;11(1):101–2.

[31] Tito de Morais A, Schneider CR, Wright WH. Diseases of high endemicity Tropical Health A Report on a Study of Needs and Resources. Washington D.C.: National Academy of Sciences—National Research Council; 1962 p. 440.

[32] Muñoz B, West SK. Blindness and visual impairment in the Americas and the Caribbean. The British journal of ophthalmology. 2002;86(5):498–504. Epub 2002/04/26. 11973241 10.1136/bjo.86.5.498 PMC1771132

[33] Muñoz B, West S. Trachoma: the forgotten cause of blindness. Epidemiologic reviews. 1997;19(2):205–17. Epub 1997/01/01. . 9494783 10.1093/oxfordjournals.epirev.a017953

[34] Silva JC. Prevencion de ceguera en America Latina: Politicas regionales de salud ocular. Arch peru oftalmol. 1999;11(1):13–23.

[35] Thylefors B, Ranson K, Négrel AD, Pararajasegaram R. Trachoma-Related Visual Loss In: Murray CJL, Lopez A. D., Mathers CD, editors. The Global Edpidemiology of Infectious Diseases. IV Geneva, Switzerland: World Health Organization; 2004 p. 301–24.

[36] Silva JC, Bateman JB, Contreras F. Eye disease and care in Latin America and the Caribbean. Survey of ophthalmology. 2002;47(3):267–74. Epub 2002/06/08. . 12052413 10.1016/s0039-6257(02)00286-2

[37] West S, Muñoz B. Tracoma en América Latina: una oportunidad para su eliminación. Biomedica: revista del Instituto Nacional de Salud. 2010;30:315–6. 21713330

[38] Anonymus. Obituary: Hideyo Noguchi M.D. N Engl J Med. 1928;198(15):829–30.

[39] Noguera JJ. Hideyo Noguchi y el tracoma (Inawashiro, Japón, 1876-Accra, Ghana, 1928). Arch Soc Esp Oftalmol. 2007;82:661–2. 17929213

[40] Wong C, Tobaru L, Tenorio A, Calderon M, Nava A, Guerra L, et al Organizacion Peruana de Lucha Contra la Ceguera (OPELUCE) y sus programas integrados de prevencion de ceguera: resultados de 15 programas rurales y 25 urbano-marginales (1983–1987). Arch peru oftalmol. 1987;2(1):21–5.

[41] Wong C, Tenorio A, Nava A, Tobaru L, Calderón M, Chea C, et al Investigación oftalmológica en los aborígenes en la selva peruana. Arch peru oftalmol. 1987;2(1):15–7.

[42] Wong C, Nava A, Tenorio A, Tobaru L, Calderón M, Chea C, et al Programa de investigación oftalmológica en los nativos de la selva peruana. Rev peru oftalmol. 1986;12(3):19–23.

[43] Wong C, Nava A, Calderon M, Tenorio A, Tobaru L, Chea C, et al Afecciones oculares y causas de ceguera en la selva peruana. Arch peru oftalmol. 1986;1(1):11–6.

[44] Wong C. Salud ocular y ceguera en el Peru: 2000–2025. Arch peru oftalmol. 2000;12:13–4.

[45] Wong C. Salud ocular y ceguera en el Peru. Arch peru oftalmol. 1996;8:3–44.

[46] Organizacion Peruana de Lucha contra la Ceguera (OPELUCE). La Historia de Opeluce en Cifras. Arch peru oftalmol. 1999;11(1):47.

[47] Organizacion Peruana de Lucha contra la Ceguera (OPELUCE). Opeluce en Acción. Arch peru oftalmol. 1999;11(1):54–6.

[48] Kuper H, Solomon AW, Buchan J, Zondervan M, Foster A, Mabey D. A critical review of the SAFE strategy for the prevention of blinding trachoma. The Lancet infectious diseases. 2003;3(6):372–81. Epub 2003/06/05. . 12781509 10.1016/s1473-3099(03)00659-5

[49] West SK. Blinding trachoma: prevention with the safe strategy. The American journal of tropical medicine and hygiene. 2003;69(5 Suppl):18–23. Epub 2003/12/25. . 14692676 10.4269/ajtmh.2003.69.18

[50] Wong J, Echegaray L. [Integrated model for the prevention of blindness based on the Peruvian Organization for the Campaign against Blindness (OPELUCE)]. Puerto Rico health sciences journal. 1993;12(2):153–6. Epub 1993/06/01. . 8210288

[51] Tenorio A, Celiz E, Kanashiro R, Wong C. Tracoma: enfermedad no cegante en aborigenes de la selva peruana. Revista del cuerpo medico. 1989;12(2):24.

[52] Paula JS, Medina NH, Cruz AA. Trachoma among the Yanomami Indians. Brazilian journal of medical and biological research = Revista brasileira de pesquisas medicas e biologicas / Sociedade Brasileira de Biofisica [et al]. 2002;35(10):1153–7. . 12424487 10.1590/s0100-879x2002001000007

[53] Taylor HR, Velasco FM, Sommer A. The ecology of trachoma: an epidemiological study in southern Mexico. Bulletin of the World Health Organization. 1985;63(3):559–67. 3876172 PMC2536438

[54] Goldschmidt P, Vanzzini Zago V, Diaz Vargas L, Espinoza Garcia L, Morales Montoya C, Peralta B, et al Chlamydia trachomatis in the conjunctiva of children living in three rural areas in Mexico. Revista panamericana de salud publica = Pan American journal of public health. 2007;22(1):29–34. . 17931485 10.1590/s1020-49892007000600004

[55] Campos JR. Prevalencia de Tracoma en una poblacion rural de Guatemala. Guatemala: Universidad Francisco Marroquin; 2003.

[56] Alves AP, Medina NH, Cruz AA. Trachoma and ethnic diversity in the Upper Rio Negro Basin of Amazonas State, Brazil. Ophthalmic epidemiology. 2002;9(1):29–34. . 11815893 10.1076/opep.9.1.29.1716

[57] Furtado JM, Lansingh VC, Carter MJ, Milanese MF, Peña BN, Ghersi HA, et al Causes of Blindness and Visual Impairment in Latin America. Survey of ophthalmology. 2012;57(2):149–77. 10.1016/j.survophthal.2011.07.002 22137039

[58] Ngondi J, Reacher M, Matthews F, Brayne C, Emerson P. Trachoma survey methods: a literature review. Bulletin of the World Health Organization. 2009;87(2):143–51. 19274367 10.2471/BLT.07.046326 PMC2636192

[59] Solomon AW, Zondervan M, Kuper H, Buchan J, Mabey D, Foster A. Trachoma Control: A Guide for Programme Managers: Stylus Pub Llc; 2006.

[60] Négrel AD, Taylor HR, West S. Guidelines for Rapid Assessment for Blinding Trachoma. WHO, editor: World Health Organization; 2001.

[61] Polack S, Brooker S, Kuper H, Mariotti S, Mabey D, Foster A. Mapping the global distribution of trachoma. Bulletin of the World Health Organization. 2005;83(12):913–9. /S0042-96862005001200013. 16462983 PMC2626493

[62] Smith JL, Haddad D, Polack S, Harding-Esch EM, Hooper PJ, Mabey DC, et al Mapping the global distribution of trachoma: why an updated atlas is needed. PLoS Negl Trop Dis. 2011;5(6):e973 10.1371/journal.pntd.0000973 21738814 PMC3125147

[63] Solomon AW, Engels D, Bailey RL, Blake IM, Brooker S, Chen JX, et al A diagnostics platform for the integrated mapping, monitoring, and surveillance of neglected tropical diseases: rationale and target product profiles. PLoS Negl Trop Dis. 2012;6(7):e1746 10.1371/journal.pntd.0001746 22860146 PMC3409112

[64] World Health Organization. Future approches to trachoma control. Report of a Global Scientific Meeting. Geneve: 1997 Contract No.: WHO/PBL/96.56.

